# It’s the Economy, Stupid: Applying (Micro)economic Principles to Microbiome Science

**DOI:** 10.1128/msystems.01033-21

**Published:** 2022-01-11

**Authors:** Nadav Kashtan, Benjamin Bushong, Johan H. J. Leveau

**Affiliations:** a Institute of Environmental Sciences, Department of Plant Pathology and Microbiology, Hebrew University of Jerusalem, Rehovot, Israel; b Department of Economics, Michigan State University, East Lansing, Michigan, USA; c Department of Plant Pathology, University of California, Davis, California, USA; University of Connecticut

**Keywords:** agent-based modeling, economics, host-microbe interactions, individual-based modeling, microeconomics, microbiome, microbiota, phyllosphere, plant-microbe interactions

## Abstract

A key challenge in microbiome science is the scale mismatch problem, which arises when the scale at which microbial communities are sampled, interrogated, and averaged is different from the scale at which individual microorganisms within those communities interact with each other and with their environment. Profiling the microbial communities in a teaspoon of soil, from a scoop of fecal matter, or along a plant leaf surface represents a scale mismatch of multiple orders of magnitude, which may limit our ability to interpret or predict species interactions and community assembly within such samples. In this Perspective, we explore how economists, who are historically and topically split along the lines of micro- and macroeconomics, deal with the scale mismatch problem, and how taking clues from (micro)economists could benefit the field of microbiomics.

## PERSPECTIVE

In the book *The Art of Clean Up: Life Made Neat and Tidy* (1), Swiss artist Ursus Wehrli shares two aerial photographs of a school’s blacktop, taken some time during class recess. The first photograph shows 60-odd scattered pupils doing typical recess things: running around, sitting together on the school steps or under the shade of a tree, or kicking a ball back and forth. The second photograph shows the same blacktop, but now the artist has “cleaned up” by making all pupils lie down on the blacktop surface, neatly arranged in histogram fashion and sorted by the color of their clothing. Next to each other, the two photographs portray the scale mismatch problem in microbiome science (43, 46) quite effectively. The cleaned-up version represents what microbiome scientists are really good at: extracting data, in classical “count and classify” style, from a theater of organismal (inter)activity, so as to allow for an approach of compare and contrast with data from other theaters in hopes of revealing patterns of presence, abundance, and cooccurrence of microbial taxa as a function of one or more properties that these theaters have in common or differ in. What gets lost in this approach is the kind of spatial context that is captured in the first of Wehrli’s photographs. Lost along with the spatial context is any chance to answer questions about which of the pupils were where and why, and who actually interacted with whom and how. If the goal were to truly understand the dynamics of pupil interactions during school recess, it would make sense to collect and compare not only coarse-grained blacktop data from different schools but also fine-grained data from different patches within single blacktops to describe and explain the whereabouts and (inter)actions of individual pupils in relation to blacktop features along dimensions that are relevant to those pupils.

**FIG 1:**
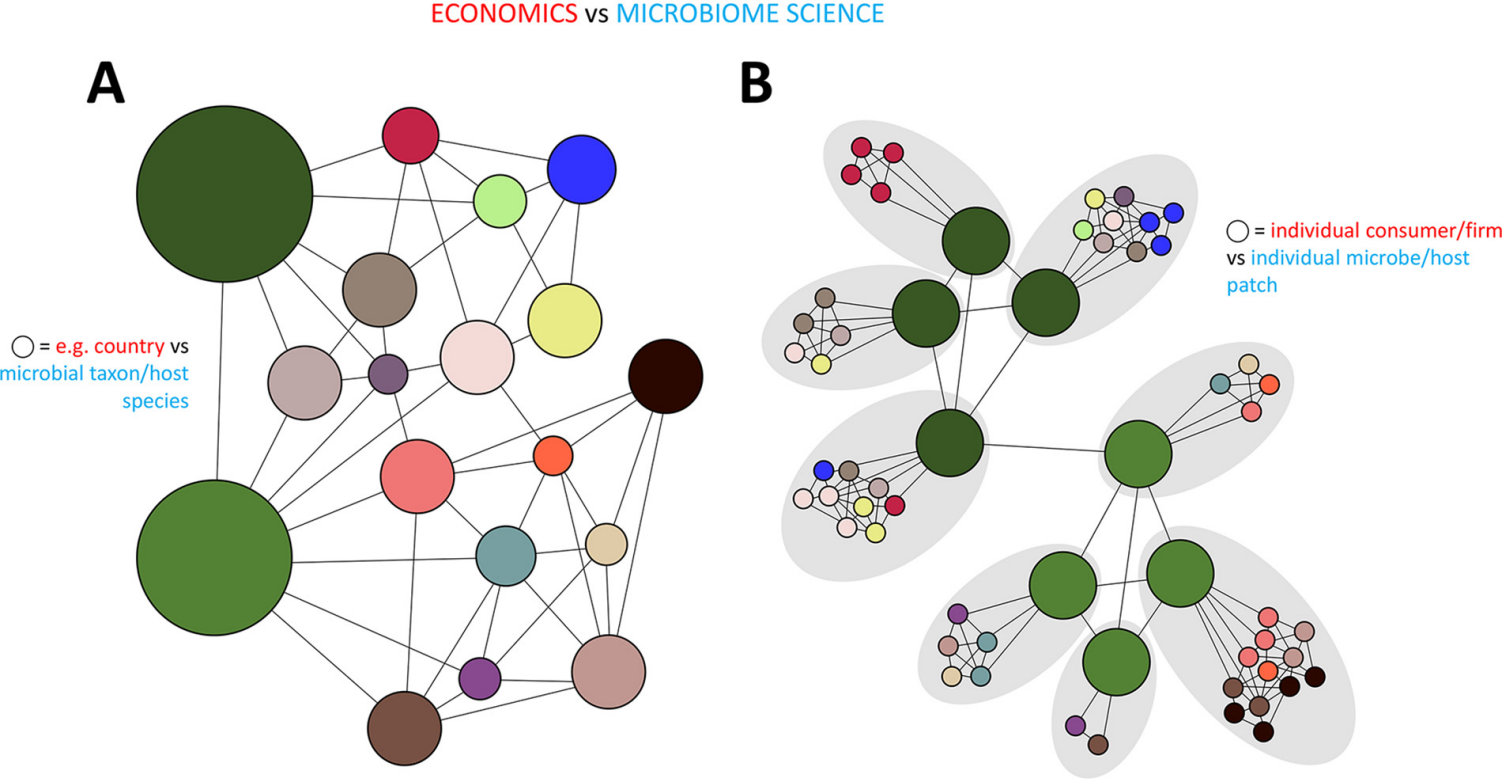
Schematic comparing the basics of macro- and microeconomics and how those might be applied to the study of microbiomes. See Box 1 and Box 2 for details.

Economists also suffer from the scale mismatch problem, and their solution has been to break up their field into two subdisciplines, which are even taught separately (2). Macroeconomics examines how countries or industries respond to large-scale changes. They measure and compare things like unemployment rates and gross national products. This can lead to valuable insights, but because macroeconomists typically examine aggregate data, they are not very well suited to elucidate the mechanisms that drive an economy (Fig. 1A and Box 1). In contrast, microeconomists study the behavior of individual firms and consumers in order to understand how and why such individuals or “agents” make economic decisions and to what degree their decision-making is determined or constrained by, for example, individual budgets, needs, or access to information. By defining what these constraints are and how they differ between individuals and along local scales, the behavior of individuals can be used to develop straightforward, tractable mathematical models of the interactions between these individuals (Fig. 1B and Box 1).

BOX 1 Figure 1 depicts a simplified comparison of macro- and microeconomics. In Fig. 1A, which offers the perspective of a macroeconomist, each circle represents some type of larger-scale economic entity, for example, a country. Each line between two circles denotes an interaction between those two entities (for example, the flow of goods, money, or information). These entities encompass multiple agents, but for the purpose of making (macro)economic predictions, the properties of all agents in each entity are often consolidated into one or more aggregate totals or average properties, and interactions between entities are calculated and predicted based on those averages. In Fig. 1B, which depicts a microeconomic view, each one of the smaller circles represents an individual agent (for example, a consumer or a firm). The color of each small circle symbolizes a property that is intrinsic to that agent, for example, the segment in the consumer market to which that individual belongs, like demographics, income, or education level. Lines between small circles correspond with interactions between individuals based on spatial, social, or other proximity (for example, membership of the same household, neighborhood, or social network). The gray ovals highlight the fact that “local” environments exist within which individuals interact with other individuals and influence each other’s decision-making. Furthermore, these environments may differ in ways that further impact or constrain the decision-making process of individuals. In this way, probing the links within a given local environment can provide critical insight into the precursors of the macroscopic activity of interest.

Microbial ecologists and microbiome scientists are not new to borrowing ideas from economists to help them solve problems. For example, the concept of market-based trade (3) has been applied to the study of microbial communities, wherein microbes “exchange” resources much like countries do in economic markets (4) and adhere to the comparative advantage principle which posits that production is allocated according to comparative rather than absolute advantage (5). Other examples include applying the notions of Pareto optimality (a key concept in economics) to explain microbial “decisions” to jointly express certain phenotypes (6), partner choice and screening host-microbe symbioses (7), and game theory to explore host-pathogen interactions in phytobiomes (8). These approaches do not typically involve the explicit consideration of microbes as individuals but instead describe interactions between distinct categories of microbes with defined traits (for example, species or functional guilds) in an idealized “world” that often lacks spatial context at the microscopic scale (Fig. 1A and Box 2). However, because variation in spatial structure is a common feature of most microbial habitats, the individual experience of the microscopic landscape by microorganisms can differ vastly along micrometer dimensions. This justifies a closer look at the promise of applying microeconomic principles to the understanding of microbiome structure and assembly (Fig. 1B and Box 2).

BOX 2 Figure 1 offers a pictorial of how macro- and microeconomic ideas apply to microbiome science. Figure 1A shows an interaction network where each circle represents a particular taxon (for example, a species or functional guild) and where a line between two circles denotes an interaction between those two taxa. In this representation, interactions typically reflect the degree of cooccurrence of taxa across a set of samples, or they may represent known taxon interactions (e.g., mutualism, parasitism, or commensalism). Different taxa are shown in different colors. In this particular representation, the large green circles might symbolize two host species that are colonized by various microbial taxa that are found on both species or are unique to either one of the species or that have a demonstrated or suspected interaction with hosts (for example, beneficial or pathogenic). Resembling a macroeconomist’s view of the world, this network representation of microbial community structure is common in microbiome science and is typically derived from taxa cooccurrence data (40), but as has been pointed out (41), cooccurrence is not necessarily evidence of ecological interactions. In contrast, the network in Fig. 1B takes a clue from microeconomists and shows individual microbial cells (small circles colored differently according to the taxon each cell belongs to) engaged in interactions with each other. These interactions are constrained to local environments (gray ovals) that are dictated by the structure of the microscopic landscape and that may differ in the availability of resources or exposure to harm. In the literature, these local microenvironments go by different names, for example, ecological neighborhoods (42), semiautonomous communities (47), and spheres of influence and perception (22). In this particular example, each gray oval also contains a larger green circle, representing the microscale portion of a host that is occupied by the individual cells in the same gray oval. Lines between dark or light green circles represent interactions between different parts of the same host. It is important to note that a spatially explicit network of interactions like the one shown in Fig. 1B is subject to changes in the microscopic landscape that can result in the continuous merging or fragmentation of local environments and in the bringing together or keeping apart of microbial cells and their translocation to other local environments. In macrobial ecology, this type of network representation is more common (44) than in microbial ecology, although there are some nice exceptions (45), not least because collecting the data to generate a graph like the one in Fig. 1B is technically challenging. The argument can be made however (see main text) that there is value in developing tools to meet this challenge in pursuit of an improved understanding of microbiome structure and assembly and the underlying mechanisms driving both.

One key principle in microeconomics is the use of agent-based models, which describe and predict agent behavior and focus on the structure and connections between individuals. Such models have yielded important insights in economics (9–11). Scaling these models from small-scale interactions to larger economies is the study of active research designed to answer the simple question of how small perturbations can echo and amplify as the unit of study goes from, say, the individual consumer or firm, to the larger economy (12, 13). Of course, a critical advantage that microeconomists have over microbiologists is that collecting data from their subjects to use in these models is relatively straightforward. While there are ways to ask single microbial cells about the experience of and response to their local environment (14), it is not a trivial task. The good news is that excellent progress is being made in the field of individual-based microbial ecology (15) which seeks to understand how individual microbes’ experiences vary, how this variation impacts the ability of individual microorganisms to survive, reproduce, coexist, and interact, and how from these microscale experiences and constraints emerge outcomes that are observable at larger spatial scales. Tools are available now to sample and interrogate at extremely small sample sizes (16). This means we can start to capture the cooccurrence and interactions of individual microbial cells along micrometer distances and to appreciate how physical, chemical, and biological features of the microscopic landscape facilitate or constrain microbial dispersal and interactions. For example, microbial proximity does not always predict microbial interaction (17), which warns against the use of artificial transects to carve up a microbial landscape, which might easily obscure long-distance interactions (such as the ball-playing kids in Wehrli’s first photograph). This further highlights the need for tools to visualize, probe, and map the microscopic landscape, in combination with spatially explicit agent-based simulation models (18, 19).

Adopting a microeconomic approach is likely to have a significant impact on the field of microbiome science. It certainly did on the field of economics. Since, at their core, economies are built on the actions, beliefs, and decisions of many individual agents interacting locally, gaining insights into these agents is a first-order concern for both micro- and macroeconomists. For instance, the fact that people prefer to consume in the present rather than the future can lead to economy-wide undersavings (20). Likewise, the constellation of psychological factors that influence consumer focus and lead to limited attention and bounded rationality bring about revisions of mainstream models of the macroeconomy (21). Thus, shedding light on these behaviors can illuminate the factors underlying economy-wide observations and improve the predictive models of macroeconomists. Here, we hypothesize that a similar individual-based approach to microbiomes will reveal novel drivers of microbiome structure and help improve the predictive models of microbiome scientists.

In support of this hypothesis, we offer a few concrete examples of how (micro)economic principles have been and continue to be applied in the study of the phyllosphere as a microbial biome. In its original definition, the term “phyllosphere” refers to plant leaf surfaces as a habitat for microorganisms (22). Typically, the phyllosphere is portrayed as an inhospitable environment, where nutrients are scarce and microbes are exposed to the dangers of desiccation and UV rays (23). Yet, leaf surfaces carry rich and diverse communities of microorganisms that are highly adapted to these conditions (24). The common microbiome science approach to leaf surfaces is quite “macroeconomic” in that microbial community structures are compared across individual or pooled leaves from different plants and plant species growing in different locations and under different conditions to reveal key insights into the role of host genotype and environment as driving factors for this microbial diversity (25). The use of gnotobiotic or synthetic communities (i.e., defined mixtures of representative species) has been an important ground-truthing tool to confirm findings about host specificity, taxon cooccurrence, and environmental impact (26).

At the scale of its microbial inhabitants however, any leaf surface is a highly heterogeneous environment, resembling a structured terrain in three dimensions, with leaf hairs, veins, and stomata dotting the microscopic landscape. Microbial colonization of the leaf surface is often patterned along this landscape: for example, the bases of leaf hairs and veins are more densely populated than other parts of the leaf surface (27). Following the microeconomic script, much of what is known about microbial life on the leaf surface comes from interrogating individual microbes about their whereabouts and experiences (28). For instance, fluorescently labeled bacterial cells revealed patterns of aggregation on the leaf surface (27), and bacterial cells from closely related taxa are more likely to be found in the same aggregate than cells from other taxa (29). Using bioreporters based on green fluorescent protein, individual cells of bacterial colonizers were questioned about their access to sugars (30), iron (31), and water (32) to reveal great variation in the availability of these resources along micrometer dimensions, even on the same leaf surface. This finding is consistent with the observed variation in reproductive success of individual bacterial immigrants to the phyllosphere: because chances of survival and producing offspring differ greatly across the leaf surface, only a subset of immigrants tends to be responsible for observed increases in bacterial population sizes (33). In phyllosphere microbiology, the concept of carrying capacity (a very macroeconomic concept, here referring to the number of microbial cells which a single leaf is able to support) has evolved toward the notion that a leaf surface represents many local carrying capacities (48) (which is a very microeconomic take on leaf surface colonization). Recent studies have demonstrated a tight interplay between leaf surface topography and free water (17), suggesting that the existence of such local carrying capacities may be linked to the presence of a fragmented landscape of small bodies of water on the surface, each harboring a subcommunity of microbial cells and species interacting locally with each other and the portion of leaf surface real estate that is covered by that body of water. Finding out what goes on in each one of those water drops (34), obtaining a more quantitative appreciation for the “reach” of microorganisms across the leaf surface (35), and linking those types of information to microbial community profiles generated from the entire leaf surface is bound to identify new types of driving factors of phyllosphere microbiome composition and assembly. There is great promise for quantifying the relative contribution of such driving factors by exploiting new and existing types of artificial theaters that mimic one or more aspects of the leaf microscopic landscape, such as wet-dry surfaces (36) and topomimetic leaves (17). Incidentally, this approach is very much analogous to that of “experimental economics,” which typically exposes subjects in a controlled environment to different situations or incentives to determine and aggregate individual behaviors and decision-making.

We believe that microbiome scientists can benefit from thinking like, talking to, or even collaborating with microeconomists. We maintain, as do others (43, 46), that a full understanding of microbiome assembly, structure, and function can only come from the appreciation of processes across a wide range of scales (37), including the smallest scale, that of the individual microbe. After all, this is the scale at which microbial cells explore, react, and cause change to their environment. At this scale, microbial agents are dealing with fundamental uncertainty, as they “make decisions” based on incomplete (e.g., only local) information. Together, these decisions create a path or history of events that cascades across the microscopic landscape and up the range of micro-, meso-, and macroscales, including the scale at which we as researchers tend to profile microbial community composition and function. Our efforts to make sense of these data and to explain patterns of microbial presence, abundance, activity, and impact have to be grounded in the notion that microbes operate at microscopic scales and that at these scales there exists substantial variation in the microscopic landscape and microbial experience. We hypothesize that it pays to know the extent of that variation and apply that knowledge to our interpretive and predictive models of microbiome assembly and structure.

Just as the predictive ability of macroeconomics is intimately tied to the insights from microeconomics, there might be no “true” microbial ecology without knowing what goes on at the micrometer scale. For microbial ecologists who are willing and keen to test this hypothesis, the seminal work by Nobel Prize-winning economist Thomas Schelling on “micromotives and macrobehaviors” offers a roadmap to expose “the relation between the behavior characteristics of the individuals who comprise some social aggregate and the characteristics of the aggregate” (38). To give just one specific example in the context of the phyllosphere microbiome, could Schelling’s model of segregation (11) explain the observed patterns of dual-species bacterial aggregates on leaf surfaces (39)? Could it guide us to first principles underlying the dynamics of aggregation and segregation of much more complex microbial communities in the phyllosphere (29)? Seeking answers to these questions starts with reaching out to potential collaborators in the field of microeconomics. We are excited about the prospect of doing so and hope that others in the field of microbiome science will do the same for their specific research questions. Who knows, maybe this even opens up ways in which microbiome scientists can “help” the field of economics, by testing economic model predictions through experimentation using microbes with different “beliefs” and “decision-making” properties as agents in heterogeneous theaters of interaction.
